# Entropy and Isokinetic Temperature in Fast Ion Transport

**DOI:** 10.1002/advs.202305065

**Published:** 2023-11-03

**Authors:** Peng Du, Hong Zhu, Artur Braun, Arthur Yelon, Qianli Chen

**Affiliations:** ^1^ University of Michigan – Shanghai Jiao Tong University Joint Institute Shanghai Jiao Tong University 800 Dong Chuan Road Shanghai 200240 China; ^2^ Key Laboratory of Interfacial Physics and Technology Shanghai Institute of Applied Physics Chinese Academy of Sciences Shanghai 201800 China; ^3^ Laboratory for High Performance Ceramics Empa. Swiss Federal Laboratories for Materials Science and Technology Dübendorf CH‐8600 Switzerland; ^4^ Département de Génie Physique and Réseau Québecois sur des Matériaux de Pointe (RQMP) Polytechnique Montréal CP 6079, Succursale C‐V Montréal QC H3C 3A7 Canada

**Keywords:** fast ionic conductors, ion transport, lattice vibration, Meyer‐Neldel rule, solid state ionics

## Abstract

Ion transport in crystalline solids is an essential process for many electrochemical energy converters such as solid‐state batteries and fuel cells. Empirical data have shown that ion transport in crystal lattices obeys the Meyer‐Neldel Rule (MNR). For similar, closely related materials, when the material properties are changed by doping or by strain, the measured ionic conductivities showing different activation energies intersect on the Arrhenius plot, at an isokinetic temperature. Therefore, the isokinetic temperature is a critical parameter for improving the ionic conductivity. However, a comprehensive understanding of the fundamental mechanism of MNR in ion transport is lacking. Here the physical significance and applicability of MNR is discussed, that is, of activation entropy‐enthalpy compensation, in crystalline fast ionic conductors, and the methods for determining the isokinetic temperature. Lattice vibrations provide the excitation energy for the ions to overcome the activation barrier. The multi‐excitation entropy model suggests that isokinetic temperature can be tuned by modulating the excitation phonon frequency. The relationship between isokinetic temperature and isokinetic prefactor can provide information concerning conductivity mechanisms. The need to effectively determine the isokinetic temperature for accelerating the design of new fast ionic conductors with high conductivity is highlighted.

## Graphical abstract

Ionic conductivities in crystal lattices often obey the Meyer‐Neldel Rule, that is, the entropy‐enthalpy compensation. In closely related materials, the ionic conductivities showing different activation energies intersect on the Arrhenius plot, at an isokinetic temperature. Here, the physical significance and applicability of is compensation effect in fast ionic conductors are discussed, and the methods for determining the isokinetic temperature.

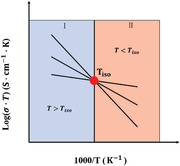

## 1 Introduction

The search for materials with high ionic mobility and diffusivity, so‐called fast ionic conductors (FICs), remains an ongoing quest. FICs are essential components for electrochemical energy conversion devices, such as solid oxide fuel cells, electrolyzers, and solid‐state batteries.^[^ ^1^, ^2^, ^3^ ^]^ The commercialization of these devices could constitute important aspects of the development of green energy. All of these applications require high ionic conductivity. To achieve these objectives, it is crucial to fully understand the peculiarities in ion transport mechanisms. The temperature dependence of ionic conductivity in solids often follows the Arrhenius law, which is characterized by constant activation energy and prefactor,^[^ ^4^ ^]^ written as:

(1) $$\;\sigma =\frac{\sigma_{0}}{T}\exp\left(-\frac{E_{a}}{k_{B}T}\right)$$

where σ is the conductivity, *T* is the temperature, σ_0_ is the prefactor, *E*_a_ is the activation energy, and *k*_B_ is the Boltzmann constant.

**Figure 1:**
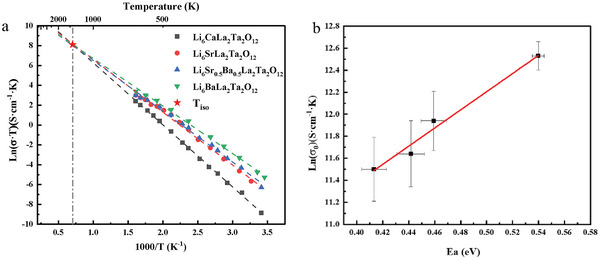
Arrhenius plot a) and Meyer‐Neldel plot b) of garnet Li_6_MLa_2_Ta_2_O_12_ (M = Ba, Ca, Sr, and Sr_0.5_Ba_0.5_) lithium ionic conductors.^[^ ^20^ ^]^ The ***T***_***iso***_ is shown as asterisk and the grey dash dot line indicates the value of ***T***_***iso***_. The solid line in (b) is the least square fit line according to Equation (2).

It is frequently assumed that decreasing the activation energy (lowering the activation barrier) of ionic conductors will improve their conductivity. However, the improvement in ionic conductivity due to the reduced activation energy may be less than expected, because the prefactor is also lower, and compensates the decrease in activation energy. In some cases, the ionic conductivity is even lower when the activation energy is reduced.^[^ ^5^ ^]^ Since the 1920s, numerous examples of such compensation have been found. This compensation effect in the prefactor is found to be proportional to *E_a_*, or to Δ*H*, the activation enthalpy.^[^ ^6^, ^7^, ^8^, ^9^, ^10^, ^11^ ^]^ This results in the intersection of the Arrhenius plots for different activation energies of related samples at an isokinetic temperature. This observation is sufficiently common to be called^[^ ^10^ ^]^ the compensation law (compensation effect^[^ ^12^ ^]^), the isokinetic law, and the MNR, for the authors who reported its observation in disordered solids.^[^ ^8^ ^]^ In particular, such an effect is frequently observed in electronic and ion conduction and in atomic and ionic diffusion.^[^ ^13^, ^14^, ^15^, ^16^, ^17^, ^18^, ^19^ ^]^ **Figure** **1**a shows an example Arrhenius plot for conductivity of garnet‐structured lithium ionic conductors Li_6_MLa_2_Ta_2_O_12_ (M = Ba, Ca, Sr, and Sr_0.5_Ba_0.5_) with variations of dopant or dopant concentration. In terms of Equation (1), with logarithm applied, we may write

(2) $$\ln\sigma_{0}=\ln\sigma_{00}+\frac{E_{a}}{k_{B}T_{\mathrm{iso}}}$$

where σ_00_ is the isokinetic prefactor, which is determined by parameters related to elementary ion hopping in FICs, and is discussed in detail in Section 6. *T_iso_* is the isokinetic temperature, and *k_B_T_iso_* is also known as Meyer‐Neldel energy, sometimes denoted as Δ_0_.

The activation energy and prefactor are determined from the slope and intercept of the Arrhenius plot, as shown in Figure 1a.The point of isokinetic temperature is also shown in Figure 1a. By fitting the prefactor as a function of activation energy using Equation (2), that is, the Meyer‐Neldel plot (M‐N plot), as shown for the garnet family Li_6_MLa_2_Ta_2_O_12_ in Figure 1b, *T_iso_* is obtained from the inverse slope of the linear least square fit to the data, as discussed below.

The MNR indicates that when the activation energy changes, the prefactor changes accordingly, compensating for the change in activation energy, reducing the improvement in conductivity. Therefore, researchers have been trying to break the limitation of the MNR^[^ ^21^ ^]^ or look for materials which do not show a compensation effect.^[^ ^22^ ^]^ However, since the ionic conductivity is affected by multiple factors related to the material structure, a comprehensive understanding of the MNR in ion transport has been challenging.

Here, we review the contribution of entropy and lattice dynamics to the ionic conductivity within the framework of the MNR. After briefly introduce the physical meaning of the MNR and the condition under which this rule is applied, we then discuss the material parameters that determine the isokinetic prefactor based on the fundamental physics of ion transport, and the effort to apply the MNR to improving the ionic conductivity by controlling the isokinetic temperature. Then we evaluate the approaches to determination of the isokinetic temperature, and the relationship between lattice vibration and isokinetic temperature. Finally, we investigate the origin of the relationship between isokinetic temperature and isokinetic prefactor and examine the mechanism of conductivity in FICs.

## 2 Activation Entropy and Isokinetic Temperature in MNR

It has been known since the work of Eyring^[^ ^23^ ^]^ in the 1920s, that a more rigorous form of the Arrhenius equation for any activated process is given by

(3) $$X=X_{0}\exp\left(\frac{-\Delta G}{k_{B}T}\right)$$

where the free energy of activation, Δ*G*, is given by

(4) ΔG=ΔH−T·ΔS

In Equation (4), Δ*H* and Δ*S* are the activation enthalpy and entropy. It has been observed that the activation enthalpy and entropy of chemical reactions often show a linear relation, the slope of which has been defined as the isokinetic temperature. But caution must be practiced to not over‐interpret the outcome of such mathematical construction.^[^ ^24^, ^25^, ^26^ ^]^ The activation entropy, Δ*S*, for movement of a carrier, electronic or ionic, called the migration entropy, is expressed as^[^ ^27^, ^28^ ^]^

(5) $$\Delta S=k_{B}\ln\left(\frac{\prod_{i\;=\;1}^{3N}v_{i}^{I}}{\prod_{i\;=\;1}^{3N-1}v_{i}^{S}}\right)$$

where $v_{i}^{I}$ and $v_{i}^{S}$ are the vibrational frequencies of the initial state and the transition state of the ion hopping process, respectively. Equation (5) shows that Δ*S* depends only upon the ratio of vibrational distribution functions of the initial and transition states.

It is now generally recognized^[^ ^10^ ^]^ that MNR occurs in closely related systems. It has recently been shown^[^ ^29^ ^]^ that it is obeyed when the free energy of the samples considered is a linear function of *T* and of one other physical variable, such as in a structure‐property relationship. We take this to be the condition for “closely related systems”. Further, $\frac{1}{T_{iso}}>0$ when Δ*H* is large compared to *k_B_T* and to the excitation energies of the system under study, and that it is an entropic effect. That is, if the process obeys MNR, there is a contribution to Δ*S* that compensates Δ*H*, and is given by:

(6) $$\Delta S_{M}=\frac{\Delta H}{T_{\mathrm{iso}}}$$

Δ*S_M_* has been called multi‐excitation entropy. The multi‐excitation entropy model^[^ ^10^ ^]^ (MEE model) suggests that, when the thermal energy is too small to provide the excitation energy for ion transport, Δ*S_M_* is associated with the collected excitations which provide the energy needed in order to overcome the barrier.

For a given Δ*H*, at low *T*, *X* is larger than it would be if such compensation did not exist. At *T_iso_*, *X* is independent of Δ*H*. If the data of Figure 1a, are extrapolated to $\frac{1}{T}=0$, we may obtain the log or ln of the experimental prefactors. If the latter is plotted as a function of *E_a_*, the inverse slope of the best linear fit yields *T_iso_* as in Figure 1b, the M‐N plot. As discussed in Section 5, this is generally a more accurate determination of *T_iso_*, than can be made from Figure 1a. Then, Δ*S_M_* can be calculated using Equation (6). However, there may be a contribution to Δ*S* that is independent of Δ*H*, which we call the change of configurational entropy, Δ*S_C_*, between the transition and initial states. Then

(7) $$\Delta S=\Delta S_{M}+\Delta S_{C}$$

It is not generally feasible to accurately calculate Δ*S_C_* from computational or experimental data. However, a comparison between Li‐ion conductors LiTi_2_(PS_4_)_3_ (LTPS) and Li_10_GeP_2_S_12_ (LGPS) suggests that a smooth energy landscape can lead to a larger entropy of the transition state.^[^ ^22^ ^]^ Fortunately, we can frequently determine the sign of Δ*S_C_*.^[^ ^10^, ^30^ ^]^ In the great majority of experimental circumstances, Δ*S_C_* is small, or positive. In a typical kinetic process, the disorder, that is, the configurational entropy, of the transition state is likely to be very little different from that of the initial state. The atoms or molecules in a chemical reaction, the mobile species in a diffusion or conduction process can move in many directions. Thus, at some *T* below *T_iso_*, either Δ*G* becomes zero, so that Arrhenius no longer applies (the process is no longer activated), or the condition *k_B_T* ≪ Δ*H* is no longer valid, so that MNR no longer applies.

In a small fraction of experiments, most notably on fast ionic conduction, as discussed below, and on relaxation of polymeric glasses,^[^ ^30^ ^]^ Δ*S_C_* is negative. Then, Δ*G* is negative at *T_iso_*, that is, the phenomenon continues to be activated, with higher Δ*H* yielding a more rapid process. If the controlling mechanism does not change, and *k_B_T* ≪ Δ*H* continues to be valid, this situation continues until a temperature, approximately:

(8) $$T=\frac{\Delta H}{\Delta S}$$

is reached, so that Δ*G* becomes zero. For entropy to be lower in the transition than in the initial state, this motion must be constrained. This is precisely the case for the two notable examples of negative Δ*S_C_* which we have cited. In glass‐forming polymers, a molecule is normally intertwined with its neighbors. For two neighboring molecules to move with respect to each other, most likely they are in a particular relative position. In each of the relaxations of a polymer glass, there are distinct values of *T_iso_* and of negative Δ*S_C_* associated with the molecular segments which move.^[^ ^30^ ^]^ For the relaxation at the highest temperature, α relaxation, for example, large portions of neighboring molecules must be close to parallel.

It is now well established that FICs, such as perovskite‐type oxides, behave as they do because of particular paths in their crystal structure,^[^ ^31^ ^]^ through which the ions can move readily. The price of this logistic advantage is their confinement, and the associated low entropy. Their conductivities above *T_iso_* may behave in a way which is quite different from that of typical semiconducting or insulating materials, whose carrier densities are activated, and carrier mobilities decrease slowly with *T*. In FICs, both maybe activated.

## 3 Applicability of MNR in Solid State Ionics

We now examine the fundamental physics of ion transport, in order to understand the effect of MNR, and especially, of *T_iso_*_,_ on ionic conductivity at *T*. The conductivity, σ, is given by:

(9) σ=cqμ

where *c* is the mobile ion concentration, *q* is the ionic charge, and *μ* is the ionic mobility.

The ionic mobility *μ* is related to the macroscopic long‐range diffusion coefficient *D*_σ_ by the Nernst‐Einstein equation:^[^ ^32^ ^]^

(10) $$\mu =\frac{qD_{\sigma }}{k_{B}T}$$

The classical model of ion diffusion in solids considers that the ion hopping events are random, that is, the ion hopping direction is independent of the previous hopping direction. This model applies when the ion concentration is low.^[^ ^33^ ^]^ When ion transport is uncorrelated and independent, one can treat *D*_σ_ as the random diffusion coefficient *D_r_*:

(11) $$D_{r}=\frac{a^{2}\nu }{b}$$

where *a* is the ion jump distance, *ν* is the ion jump frequency for successful jumps which leads to macroscopic diffusion, *b* is a geometric factor, for 1D, 2D, or 3D diffusion, *b* is 2, 4, or 6 respectively.

The ion jump frequency ν is described by:

(12) $$\nu =\nu_{0}\exp\left(-\frac{\Delta G}{k_{B}T}\right)=\nu_{0}\exp\left(\frac{\Delta S}{k_{B}}\right)\exp\left(-\frac{\Delta H}{k_{B}T}\right)$$

where ν_0_ is the attempt frequency which includes both the successful jumps and unsuccessful jumps.

According to Equation (9) to Equation (12), the complexity of temperature‐dependent ionic conductivity σ can be mathematically illustrated by:^[^ ^34^ ^]^

(13) $$\sigma \left(T\right)=\frac{1}{b}\frac{cq^{2}}{k_{B}T}a^{2}\upsilon_{0}\exp\left(\frac{\Delta S}{k_{B}}\right)\exp\left(-\frac{\Delta H}{k_{B}T}\right)$$

where the enthalpy Δ*H* is also called activation energy (denoted as *E_a_*). Comparing Equation (1), and Equation (13), the prefactor is written as:^[^ ^5^ ^]^

(14) $$\sigma_{0}=\frac{1}{b}\frac{cq^{2}}{k_{B}}a^{2}\upsilon_{0}\exp\left(\frac{\Delta S}{k_{B}}\right)$$

From Equation (14), one may see that, in addition to the entropy, the prefactor is affected by many parameters, including the mobile ion concentration, ion jump distance, and attempt frequency. Therefore, it is worth noting that MNR applies only to “closely related systems”, when the material candidates have similar composition and structure, so that other parameters do not differ much between different samples or for variable measurement parameters, as discussed in the literature on chemical reactions^[^ ^24^, ^25^, ^35^ ^]^ and in the previous and following sections. However, when the ion concentration changes, the defect formation energy may also vary, causing changes in the activation energy. The variation in ion concentration adds difficulties to the determination of *T_iso_*, and may be one reason for the scattering of data. Therefore, we propose that *T_iso_* can be more rigorously determined using the diffusion coefficients instead of ionic conductivities, when the influence of concentration is excluded.

## 4 Improving the Ionic Conductivity According to the MNR

Reducing the activation energy has been considered to be an effective method for improving ionic conductivity. However, the MNR shows that lower activation energy is not always related to high ionic conductivity. Here, we consider how activation energy determines the ionic conductivity according to MNR.

**Figure 2:**
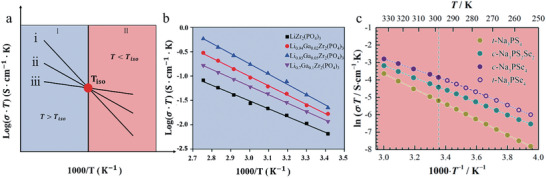
a) The role of ***T***_***iso***_ in ionic conductivity. Region I (blue) and Region II (pink) represent the cases of measurement temperature larger and smaller than ***T*_iso_**. The lines i, ii, and iii represent three conductivity lines with activation energies and prefactors decreasing sequentially. b) the lithium‐ion conductivity of Li_1‐3_ *_x_* Ga*_x_* Zr_2_(PO_4_)_3_ (*x* = 0, 0.02, 0.05, 0.1). Reproduced with permission.^[^ ^36^ ^]^ Copyright 2021, The Royal Society of Chemistry. c) The sodium ionic conductivity of Na_3_PS_4‐_ *_x_* Se*_x_* (*x* = 0, 2, 4). Reproduced with permission.^[^ ^37^ ^]^ Copyright 2018, American Chemical Society.

Because of the entropy‐enthalpy compensation suggested by the MNR, we can use *E_a_* to replace Δ*S_M_* to formally describe the ionic conductivity. That is, combining Equation (2), Equation (6), Equation (7), and Equation (13), the conductivity becomes:

(15) $$\sigma \left(T\right)=\frac{1}{b}\frac{cq^{2}}{k_{B}T}a^{2}\upsilon_{0}\exp\left(\frac{\Delta S_{C}}{k_{B}}\right)\exp\left[\left(\frac{E_{a}}{k_{B}}\right)\left(\frac{T-T_{\mathrm{iso}}}{T_{\mathrm{iso}}T}\right)\right]$$

where *T* is the measurement temperature, or the operating temperature. Equation (15) suggests that the argument $\left(\frac{E_{a}}{k_{B}}\right)\left(\frac{T-T_{iso}}{T_{iso}T}\right)$ in the exponent may be considered to be an indicator for modulating the material conductivity. If *T* > *T_iso_*, the measured conductivity falls in Region I in **Figure** **2**a. That is, the ionic conductivity is higher when *E_a_* is larger. When *T* < *T_iso_* and the measured conductivity is located in Region II in Figure 2b, it is necessary to decrease *E_a_* to improve the ionic conductivity. When *T* = *T_iso_*, the conductivity is independent of *E_a_*.

In Figure 2 we show examples of the behavior described above. In Figure 2b, the conductivity of Na_3_PS_4‐_ *_x_* Se*_x_* decreases with the increase of *E_a_*, whereas that of Li_1‐3x_Ga_x_Zr_2_(PO_4_)_3_ (LGZP) increases with the increase of *E_a_* in Figure 2c. The *T_iso_* of LGZP^[^ ^36^ ^]^ and Na_3_PS_4‐_ *_x_* Se*_x_* ^[^ ^37^ ^]^ are presented in **Table** **1**. According to the above discussion, the conductivity of LGZP and Na_3_PS_4‐_ *_x_* Se*_x_* should fall in Region I and Region II of Figure 2a, respectively. This is confirmed by the measured conductivity, as shown in Figure 2b,c. The proton conductivity of BaZr_0.9_Y_0.1_O_3_ under high pressure^[^ ^38^ ^]^ shows the same behavior as in the Na‐ion conductivity of Na_3_PS_4‐_ *_x_* Se*_x_*. More data from various lithium‐ion and proton conductors are presented in Tables S1 and S2 (Supporting Information). For most of the materials investigated, *T_iso_* is higher than the measurement temperature.

**Table 1:** The measurement temperature ***T***, isokinetic temperature ***T***_***iso***_, and ***T vs***. *** T***_***iso***_ of Li_1‐3_ *_x_* Ga*_x_* Zr_2_(PO_4_)_3_^[^ ^37^ ^]^ and Na_3_PS_4‐_ *_x_* Se*_x_* (*x* = 0, 2, 4).^[^ ^36^ ^]^

Material	Operating temperature *T* [K]	*T_iso_* [K]	*T* *vs*. *T_iso_*
Li_1‐3_ *_x_* Ga*_x_* Zr_2_(PO_4_)_3_	293–363	224.9	*T* > *T_iso_*
Na_3_PS_4‐_ *_x_* Se*_x_* (*x*=0, 2, 4)	250–330	396.7	*T* < *T_iso_*

The activation energy can be affected by multiple factors including crystal symmetry, defects, and lattice softness.^[^ ^34^ ^]^ For related material systems, the activation energy is lower when the crystal structure exhibits higher symmetry and less disorder. A softer lattice is generally considered to be related to lower activation energy.^[^ ^21^ ^]^ In practice, the activation energy can be tuned by doping,^[^ ^39^ ^]^ applying strain,^[^ ^40^ ^]^ by modifying the vibration frequency,^[^ ^21^ ^]^ and the density of grain boundaries.^[^ ^41^ ^]^

Notably, interesting results have been found in recent investigations whose objective was to reduce the activation energy. For instance, in the materials that manifest mobile ion disordering, the frustration in the LTPS framework enlarges the ionic jump distances compared to LGPS.^[^ ^22^ ^]^ As a result, LTPS exhibits a larger prefactor but a lower activation energy, which deviates from the MNR. Thus, the disorder in the material offers an alternative approach to significantly increasing the ionic conductivity.

## 5 Determination and Physical Significance of Isokinetic Temperature in Fast Ion Conductors: The Role of Lattice Vibrations

**Figure 3:**
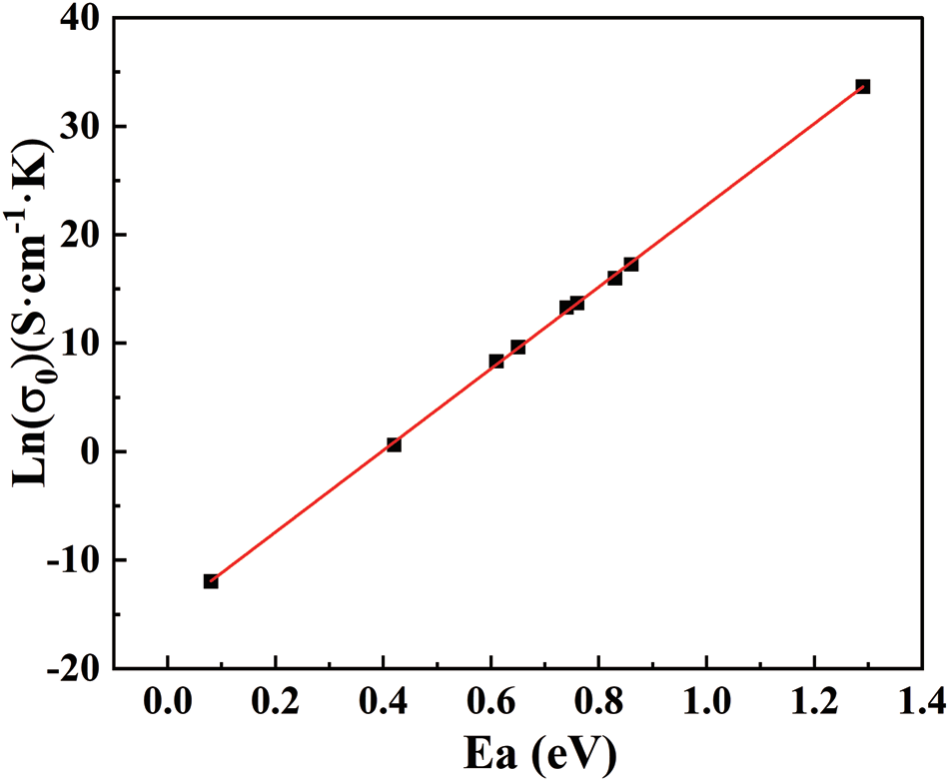
Meyer‐Neldel plot of Li_6.4_La_3_Zr_1.4_Ta_0.6_O_12_ under variable pressure.^[^ ^42^ ^]^

In Section 3, we have suggested that isokinetic temperature is affected by the material structure, lattice parameters, and composition. All these factors vary when the dopant concentration changes, thus makes the determination of isokinetic temperature challenging. Most often, the investigation of MNR of a property of a family of similar materials involves the preparation of a material by different techniques, or of similar composition, for example, by element substitution and doping.^[^ ^17^, ^39^ ^]^ Conduction in FICs may also be investigated using a single sample by imposing strain in the material (e.g., with pressure^[^ ^38^ ^]^). For instance, **Figure** **3** shows a M‐N plot demonstrating the strain‐induced variation in *E_a_* and σ_0_ under high compressive strain.^[^ ^42^ ^]^ Then *T_iso_* is calculated from Equation (2) according to experimental data.

It is evident from Figure 3 that under these circumstances, of a single material under varying pressure, MNR is rigorously obeyed, as predicted by Sapunov.^[^ ^35^ ^]^ In contrast, in Figure 1b, in which garnets containing different ions are compared, the criterion of “closely related systems” is not completely satisfied. When tuning the material composition, the adjustment in lattice parameters leads to changes in migration entropy. We cannot use MNR to determine the properties of these materials. However, it can still be used as a rule of thumb to suggest that such systems may be expected to exhibit similar values of *T_iso_*_._^[^ ^17^, ^42^, ^43^ ^]^

A recent molecular dynamics (MD) study of MNR in atomic diffusion in simple metals^[^ ^44^ ^]^ strongly suggests that the phenomenon is quite complicated, with substantial changes in vibrational densities of states between the initial and the transition state during diffusion, and that this provides the entropy for the compensation. Density functional theory and other first principles computational methods can only obtain the entropy, *S*, and activation energy *E_a_*, of the initial state, but not Δ*S_M_*.^[^ ^34^, ^42^ ^]^ Therefore, isokinetic temperature cannot be calculated from such theories, for now. To date, there have been no MD studies of MNR in ion conduction. Thus, we rely upon simple models or experiments to clarify the situation.

Within the simple, phenomenological, MEE model,^[^ ^10^, ^45^ ^]^ inspired by microscopic models for particular processes, a prediction for *T_iso_* has been proposed. It assumes that one excitation of the system is most strongly coupled to that process which takes place, when Δ*H* is large, a number of these excitations must be accumulated to overcome the barrier. This concentration results in Δ*S_M_*.^[^ ^10^ ^]^ For optical phonons it is proposed that:

(16) $$k_{B}T_{iso}=\frac{hv}{ln\kappa }$$

where *h* is Planck's constant, *hv* is the excitation energy, and *κ* is a coupling constant. It has been suggested that *ln* *κ* for polaronic materials, particularly ionic polarons, is related to the characteristic phonon occupation,^[^ ^46^ ^]^ that is, the phonon number in a specific excitation state of vibrational mode.

The MEE model prediction^[^ ^10^ ^]^ for *T_iso_* of a material, in which the only excitations are acoustic phonons, is different from Equation (16). In that case, it does not predict a particular frequency within the broad spectrum, for a situation such as that considered in ref.[44]. In experiments concerning a number of phenomena, it has been possible to identify excitations which satisfy Equation (16), assuming^[^ ^10^ ^]^ that *ln*κ ranges between 0.5 and 2. These include studies of chemical reactions,^[^ ^47^ ^]^ where the excitations are those of molecular vibrations; relaxations,^[^ ^48^ ^]^ where they are the energies of the relaxing entities, conduction of FICs,^[^ ^17^, ^18^, ^43^ ^]^ where they are optical phonons.

**Figure 4:**
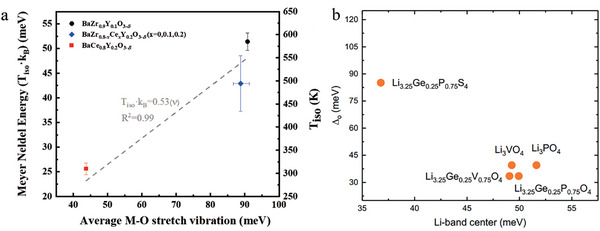
a) The relationship between Meyer–Neldel energy or isokinetic temperature and the average M‐O stretch vibration 〈ν〉 for BaZr_0.9_Y_0.1_O_3−δ_, BaZr_0.8−x_Ce_x_Y_0.2_O_3−δ_ (*x* = 0, 0.1, 0.2), and BaCe_0.8_Y_0.2_O_3−δ_. Reproduced with permission.^[^ ^17^ ^]^ Copyright 2021, Wiley‐VCH. b) The relationship between Meyer–Neldel energy (***Δ***_0_) and Li‐band center for Li_3.25_Ge_0.25_P_0.75_S_4_, Li_3.25_Ge_0.25_V_0.75_O_4_, Li_3_VO_4_, Li_3_PO_4,_ and Li_0.325_Ge_0.25_P_0.75_O_4_. Reproduced with permission.^[^ ^49^ ^]^ Copyright 2018, American Chemical Society.

It is still not clear whether Equation (16) is widely applicable. For example, in Y‐doped BaMO_3_ (M = Zr or Ce) proton conductors, the relation between isokinetic temperature and average M‐O stretch vibration follows Equation (16), as shown in **Figure** **4**a.^[^ ^17^ ^]^ In contrast, in lithium superionic conductors (LISICON) and olivine compounds, the phonon band center decreases with the increase of Meyer‐Neldel energy, as illustrated in Figure 4b.^[^ ^49^ ^]^ It is possible that the excitations responsible for *T_iso_* are not Li phonons in these lithium conductors. However, recent work on garnet‐type lithium conductors using isotope substitution have demonstrated that lower lithium vibration frequency corresponds to higher ionic conductivity.^[^ ^50^ ^]^ Clearly, further investigation as to whether Equation (16) is universally applicable to FICs is needed. If it is, it is important to identify the phonons which contribute the excitation that determines the isokinetic temperature. One promising experimental method to perform this task could be nuclear resonant vibration spectroscopy (NRVS).^[^ ^51^ ^]^ New developments in synchrotron based x‐ray methods permit the determination of element‐specific vibration spectra, provided the element is available as a Mössbauer active isotope.^[^ ^51^ ^]^

## 6 Relationship between Isokinetic Temperature and Isokinetic Prefactor

**Figure 5:**
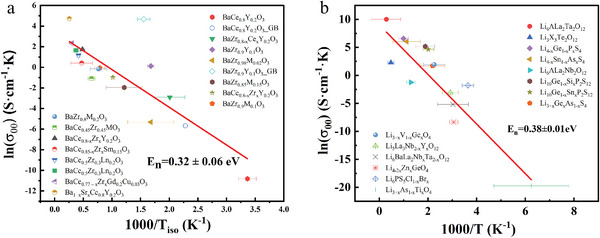
Correlation between isokinetic prefactor ***σ***_**00**_ and isokinetic temperature ***T***_***iso***_ for various compositions of perovskite‐type proton conductors a), and lithium‐ion conductors b).^[^ ^55^, ^56^, ^57^, ^58^, ^59^, ^60^, ^61^, ^62^, ^63^, ^64^, ^65^ ^]^ The solid lines are the fit according to Equation (17). In (a), the solid, hollow, and semi‐solid symbols correspond to the grain, grain boundary, and total conductivity. (All the relevant data obtained from literature which is found in Tables S1 and S2, Supporting Information).

Over the past several decades, it has been observed^[^ ^10^ ^]^ that for some families of electronic and ionic conductors obeying MNR, there is a correlation, that is, an approximate relationship between the isokinetic prefactor, σ_00_ and the isokinetic temperature *T_iso_*,^[^ ^10^, ^17^, ^43^, ^52^, ^53^ ^]^

(17) $$\ln\sigma_{00}=\ln\sigma_{00}^{{}^{\prime }}-\frac{E_{n}}{k_{B}T_{\mathrm{iso}}}$$

where $\sigma_{00}^{\prime }$ and *E_n_* are empirical constants, discussed below. From Equation (16) we see that *T_iso_* is inversely proportional to *κ*, the coupling to excitations. On this basis, it has been proposed^[^ ^10^, ^43^ ^]^ that positive *E_n_* corresponds to polaronic conduction, while negative *E_n_* corresponds to trap‐limited conduction. This has led to the identification of polaronic conduction in numerous materials. These include electronic conduction in chalcogenide glasses,^[^ ^54^ ^]^ proton conductivity in minerals,^[^ ^19^ ^]^ and ionic conductivity in perovskite‐type oxides.^[^ ^17^ ^]^ **Figure** **5** shows other examples from the literature.^[^ ^55^, ^56^, ^57^, ^58^, ^59^, ^60^, ^61^, ^62^, ^63^, ^64^, ^65^ ^]^

Figure 5a shows the isokinetic prefactor σ_00_ as a function of the reciprocal of isokinetic temperature *T_iso_* for some perovskite‐type proton conductors. Neutron scattering experiments have also confirmed that for perovskite proton conductors, the proton jump time follows a polaron model.^[^ ^66^, ^67^ ^]^ Figure 5b shows a similar relationship for the LGPS family Li‐ion conductors with variable compositions. Note that the data points scatter because the criterion of closely related system is not completely satisfied, for instance, the mobile ion concentration, attempt frequency, or other parameters change. Now, let us consider the significance of Equation (17) and the physical meaning of $\sigma_{00}^{\prime }$ and *E_n_*.

Applying Equation (2), Equation (6), and Equation (7) to Equation (14), yields

(18) $$\sigma_{00}=\frac{1}{b}\frac{cq^{2}}{k_{B}}a^{2}\upsilon_{0}\exp\left(\frac{\Delta S_{C}}{k_{B}}\right)$$

Therefore, in random and uncorrelated ion hopping processes, the isokinetic parameter, σ_00_, is determined by the ion concentration, jump distance, attempt frequency, and Δ*S_C_*. As discussed in Section 2, Δ*S_C_* is positive or small for most properties of most materials. However, as shown in Figure 2, it is negative and non‐negligible for conductivity of FICs. As we may see, σ_00_ depends exponentially upon Δ*S_C_*. There is no reason to expect the linear terms in Equation (8) to exhibit large changes from one member of a family to another. This leads us to suggest that, for FICs:

(19) $$\sigma_{00}^{{}^{\prime }}=\frac{1}{b}\frac{cq^{2}}{k_{B}}a^{2}\upsilon_{0}$$

and *E_n_* is expressed by:

(20) $$E_{n}=-T_{iso}\Delta S_{C}$$

Therefore, *E_n_* is related to the change of configurational entropy between initial and translational states, Δ*S_C_*. It has been suggested that the value of *E_a_* compared to *E_n_* can determine the direction to modulate the isokinetic temperature in order to tune the ionic conductivity.^[^ ^17^ ^]^ and thus *E_n_* is called the critical energy of materials.^[^ ^17^ ^]^

Nevertheless, the extent to which the model we have proposed here may apply to other materials, including isotropic ionic conductors, is not evident. As pointed out in Section 3, most properties which obey MNR exhibit positive or very small Δ*S_C_*_,_ which should then not determine the sign of *E_n_*_._ However, in Figure 5, the calculated *E_n_* according to Equation (17) is 0.32 ± 0.06 eV for perovskite‐type proton conductors and 0.38 ± 0.01 eV for Li‐ion conductors, respectively, indicating that *E_n_* differs among different types of ionic conductors and can also be large. This also requires further investigation.

## 7 Conclusion

It has been shown that MNR is the result of entropy‐enthalpy compensation, and it has been proposed that multi‐excitation entropy, MEE compensates the lack of thermal energy to overcome the energy barrier to ion transport in FICs. Within this framework, the dependence of Arrhenius prefactor on the activation energy may be applied to closely related systems, in which the material parameters, including mobile ion concentration, ion jump distance, and attempt frequency, do not differ greatly. Applying these constraints, we have investigated the roles of entropy, of isokinetic temperature, *T_iso_*, and the contribution of lattice dynamics in the ion transport processes in FICs.

The value of operating temperature *T* compared to *T_iso_* determines the strategy for improving the conductivity by tuning the activation energy. When *T* < *T_iso_*, the conductivity can be improved by decreasing the activation energy. When *T* > *T_iso_*, it can be improved by increasing it. Since MNR applies to closely related systems, caution must be taken to determine its value. *T*_*iso*_ may be determined experimentally, using an Arrhenius plot or a M‐N plot, using Equation (2). The pressure‐tuning method is an effective approach for measuring *T_iso_* in FICs. Controlling its value remains challenging, due to the lack of theoretical models. The MEE model suggests that *T_iso_* is proportional to the excitation phonon frequency, given by Equation (16). The general validity of this prediction, in particular its applicability to FICs continues to be investigated.

Finally, we have proposed that for FICs, the critical energy *E_n_*, determined from the relationship between isokinetic prefactor σ_00_ and isokinetic temperature *T_iso_*, is related to the configurational entropy change between the initial and transition sites. A positive *E_n_* suggests that the charge carriers behave as polarons. The new understanding will add insights to develop new FICs.

## Conflict of Interest

The authors declare no conflict of interest.

## Supporting information

Supporting Information

## References

[1] D. Han , X. Liu , T. S. Bjørheim , T. Uda , Adv. Energy Mater. 2021, 11, 2003149.

[2] H. An , D. Shin , S. M. Choi , J.‐H. Lee , J.‐W. Son , B.‐K. Kim , H. J. Je , H.‐W. Lee , K. J. Yoon , J. Korean Ceram. Soc. 2014, 51, 271.

[3] B. Zhu , L. Fan , P. Lund , Appl. Energy 2013, 106, 163.

[4] S. Arrhenius , Z. Phys. Chem. 1889, 4, 96.

[5] M. A. T. Marple , B. G. Aitken , S. Kim , S. Sen , Chem. Mater. 2018, 30, 5896.

[6] F. H. Constable , Proc. R. soc. Lond. Ser. A‐Contain. Pap. Math. Phys. Character 1925, 108, 355.

[7] G. Schwab , Z. Phys. Chem. 1929, 5, 406.

[8] W. Meyer , H. Neldel , Phys. Z. 1937, 38, 1014.

[9] D. Emin , Phys. Rev. Lett. 2008, 100, 166602. 18518230 10.1103/PhysRevLett.100.166602

[10] A. Yelon , B. Movaghar , R. Crandall , Rep. Prog. Phys. 2006, 69, 1145.

[11] M. J. Polissar , J. Am. Chem. Soc. 1930, 52, 956.

[12] C. L. Perrin , I. Agranat , A. Bagno , S. E. Braslavsky , P. A. Fernandes , J.‐F. Gal , G. C. Lloyd‐Jones , H. Mayr , J. R. Murdoch , N. S. Nudelman , L. Radom , Z. Rappoport , M.‐F. Ruasse , H.‐U. Siehl , Y. Takeuchi , T. T. Tidwell , E. Uggerud , I. H. Williams , Pure Appl. Chem. 2022, 94, 353.

[13] S. Kadkhodaei , A. van de Walle , J. Chem. Phys. 2019, 150, 144105. 30981228 10.1063/1.5086746

[14] J. N. Lalena , D. A. Cleary , O. B. H. Duparc , Principles of Inorganic Materials Design, John Wiley & Sons, Hoboken, NJ 2020.

[15] M. Li , C. Wang , Z. Chen , K. Xu , J. Lu , Chem. Rev. 2020, 120, 6783. 32022546 10.1021/acs.chemrev.9b00531

[16] Y.‐Y. Lin , A. X. B. Yong , W. J. Gustafson , C. N. Reedy , E. Ertekin , J. A. Krogstad , N. H. Perry , Curr. Opin. Solid State Mater. Sci. 2020, 24, 100875.

[17] P. Du , N. Li , X. Ling , Z. Fan , A. Braun , W. Yang , Q. Chen , A. Yelon , Adv. Energy Mater. 2021, 12, 2102939.

[18] K. Shimakawa , M. Aniya , Monatsh. Chem. 2013, 144, 67.

[19] A. G. Jones , Geochem. Geophys. Geosyst. 2014, 15, 2616.

[20] R. Murugan , V. Thangadurai , W. Weppner , J. Electrochem. Soc. 2007, 155, A90.

[21] R. S. Sokseiha Muy , Y. Shao‐Horn , W G. Zeier , Adv. Energy Mater. 2020, 11, 2002787.

[22] D. Di Stefano , A. Miglio , K. Robeyns , Y. Filinchuk , M. Lechartier , A. Senyshyn , H. Ishida , S. Spannenberger , D. Prutsch , S. Lunghammer , D. Rettenwander , M. Wilkening , B. Roling , Y. Kato , G. Hautier , Chem 2019, 5, 2450.

[23] S. Glasstone , K. J. Laidler , H. Eyring , The Theory of Rate Processes; the Kinetics of Chemical Reactions, Viscosity, Diffusion, Electrochemical Phenomena, McGraw‐Hill Book Company, New York 1941.

[24] J. E. Leffler , J. Org. Chem. 1955, 20, 1202.

[25] O. Exner , Nature 1964, 201, 488.

[26] R. C. Petersen , J. Org. Chem. 1964, 29, 3133.

[27] T. Krauskopf , C. Pompe , M. A. Kraft , W. G. Zeier , Chem. Mater. 2017, 29, 8859.

[28] R. Chen , Z. Xu , Y. Lin , B. Lv , S.‐H. Bo , H. Zhu , ACS Appl. Energy Mater. 2021, 4, 2107.

[29] A. Yelon , E. Sacher , W. Linert , Phys. Chem. Chem. Phys. 2012, 14, 8232. 22555217 10.1039/c2cp40618g

[30] J.‐P. Crine , IEEE Trans. Dielectr. Electr. Insul. 2017, 24, 3750.

[31] F. M. Draber , C. Ader , J. P. Arnold , S. Eisele , S. Grieshammer , S. Yamaguchi , M. Martin , Nat. Mater. 2020, 19, 338. 31873227 10.1038/s41563-019-0561-7

[32] A. Cooper , Mass transport phenomena in ceramics, Springer Science & Business Media, New York 2012.

[33] H. Mehrer , Diffusion in solids: fundamentals, methods, materials, diffusion‐controlled processes, Springer Science & Business Media, New York 2007.

[34] Y. Gao , A. M. Nolan , P. Du , Y. Wu , C. Yang , Q. Chen , Y. Mo , S. H. Bo , Chem. Rev. 2020, 120, 5954. 32347715 10.1021/acs.chemrev.9b00747

[35] V. N. Sapunov , E. A. Saveljev , M. S. Voronov , M. Valtiner , W. Linert , Thermo 2021, 1, 45.

[36] S. Duan , C. Huang , M. Liu , Z. Cao , X. Tian , S. Hou , J. Li , B. Huang , H. Jin , J. Mater. Chem. A 2021, 9, 7817.

[37] T. Krauskopf , S. Muy , S. P. Culver , S. Ohno , O. Delaire , Y. Shao‐Horn , W. G. Zeier , J. Am. Chem. Soc. 2018, 140, 14464. 30284822 10.1021/jacs.8b09340

[38] Z. Fan , N. Li , P. Du , W. Yang , Q. Chen , J. Phys. Chem. C 2020, 124, 22376.

[39] D. Han , K. Goto , M. Majima , T. Uda , ChemSusChem 2021, 14, 614. 33150740 10.1002/cssc.202002369

[40] A. Fluri , A. Marcolongo , V. Roddatis , A. Wokaun , D. Pergolesi , N. Marzari , T. Lippert , Adv. Sci. 2017, 4, 1700467. 10.1002/advs.201700467 PMC5737104 29270353

[41] L. P. Wendler , K. Ramos , D. M. P. F. Souza , Ceram. Int. 2019, 45, 19120.

[42] Y. Gao , N. Li , Y. Wu , W. Yang , S. H. Bo , Adv. Energy Mater. 2021, 11, 2100325.

[43] A. Braun , Q. Chen , A. Yelon , Chimia 2019, 73, 936. 31753075 10.2533/chimia.2019.936

[44] S. Gelin , A. Champagne‐Ruel , N. Mousseau , Nat. Commun. 2020, 11, 3977. 32770040 10.1038/s41467-020-17812-2 PMC7414111

[45] A. Yelon , B. Movaghar , H. M. Branz , Phys. Rev. B Condens. Matter 1992, 46, 12244. 10003136 10.1103/physrevb.46.12244

[46] T. Bernges , R. Hanus , B. Wankmiller , K. Imasato , S. Lin , M. Ghidiu , M. Gerlitz , M. Peterlechner , S. Graham , G. Hautier , Y. Pei , M. R. Hansen , G. Wilde , G. J. Snyder , J. George , M. T. Agne , W. G. Zeier , Adv. Energy Mater. 2022, 12, 2200717.

[47] W. Linert , R. Jameson , Chem. Soc. Rev. 1989, 18, 477.

[48] R. A. Masut , J. Appl. Phys. 2022, 132, 084901.

[49] S. Muy , J. C. Bachman , H.‐H. Chang , L. Giordano , F. Maglia , S. Lupart , P. Lamp , W. G. Zeier , Y. Shao‐Horn , Chem. Mater. 2018, 30, 5573.

[50] Y. Gao , J. Huang , J. Cheng , S.‐H. Bo , Sci. China Chem. 2023, 66, 768.

[51] H. Wang , A. Braun , S. P. Cramer , L. B. Gee , Y. Yoda , Crystals 2021, 11, 909. 10.3390/cryst11080909 PMC9109880 35582460

[52] J. C. Wang , Y. F. Chen , Appl. Phys. Lett. 1998, 73, 948.

[53] A. Yelon , MRS Adv 2017, 2, 425.

[54] F. Abdel‐Wahab , A. Yelon , J. Appl. Phys. 2013, 114, 023707.

[55] S. Wang , F. Zhao , L. Zhang , K. Brinkman , F. Chen , J. Alloys Compd. 2010, 506, 263.

[56] D. A. Medvedev , E. V. Gorbova , A. K. Demin , B. D. Antonov , Russ. J. Electrochem. 2011, 47, 1404.

[57] N. Danilov , E. Pikalova , J. Lyagaeva , B. Antonov , D. Medvedev , A. Demin , P. Tsiakaras , J. Power Sources 2017, 366, 161.

[58] J. Lyagaeva , D. Medvedev , E. Filonova , A. Demin , P. Tsiakaras , Scripta Mater 2015, 109, 34.

[59] D. Han , N. Hatada , T. Uda , J. Electrochem. Soc. 2016, 163, F470.

[60] P. Sawant , S. Varma , B. N. Wani , S. R. Bharadwaj , Int. J. Hydrogen Energy 2012, 37, 3848.

[61] T. Krauskopf , S. P. Culver , W. G. Zeier , Chem. Mater. 2018, 30, 1791.

[62] Z. Jiang , Z. Li , X. Wang , C. Gu , X. Xia , J. Tu , ACS Appl. Mater. Interfaces 2021, 13, 30739. 34169722 10.1021/acsami.1c07947

[63] Y. Kato , R. Saito , M. Sakano , A. Mitsui , M. Hirayama , R. Kanno , J. Power Sources 2014, 271, 60.

[64] J. Gao , X. Sun , C. Wang , Y. Zhang , L. Yang , D. Song , Y. Wu , Z. Yang , T. Ohsaka , F. Matsumotoc , J. Wu , ChemElectroChem 2022, 9, 202200507.

[65] Y. Sun , K. Suzuki , K. Hara , S. Hori , T. A. Yano , M. Hara , M. Hirayama , R. Kanno , J. Power Sources 2016, 324, 798.

[66] A. Braun , Q. Chen , Nat. Commun. 2017, 8, 15830. 28613274 10.1038/ncomms15830 PMC5474746

[67] P. Du , Q. Chen , Z. Fan , H. Pan , F. G. Haibach , M. A. Gomez , A. Braun , Commun. Phys. 2020, 3, 200.

