# Supplementary material for: Entropy and Isokinetic Temperature in Fast Ion Transport
Source: Adv Sci (Weinh). 2023 Nov 3;11(2):2305065. doi: 10.1002/advs.202305065 (PMC10787107; doi:10.1002/advs.202305065)
Supplement: Supplementary file 1 — Supporting Information [file ADVS-11-2305065-s001.pdf]

ADVANCED SCIENCE Open Access

Supporting Information

for *Adv. Sci.*, DOI 10.1002/advs.202305065

Entropy and Isokinetic Temperature in Fast Ion Transport

*Peng Du, Hong Zhu, Artur Braun, Arthur Yelon and Qianli Chen**

Supporting Information

Entropy and Isokinetic Temperature in Fast Ion Transport

*Peng Du, Hong Zhu, Artur Braun, Arthur Yelon, and Qianli Chen**

Peng Du, Hong Zhu, Qianli Chen

University of Michigan – Shanghai Jiao Tong University Joint Institute, Shanghai Jiao Tong University, 800 Dong Chuan Road, Shanghai, 200240, China

E-mail: qianli.chen@sjtu.edu.cn

Peng Du

Key Laboratory of Interfacial Physics and Technology, Shanghai Institute of Applied Physics, Chinese Academy of Sciences, Shanghai 201800, China

Artur Braun

Laboratory for High Performance Ceramics, Empa. Swiss Federal Laboratories for Materials Science and Technology, CH-8600 Dübendorf, Switzerland

Arthur Yelon

Département de Génie Physique, Polytechnique Montréal, CP 6079, Succursale C-V, Montréal, QC, H3C 3A7, Canada, and Réseau Québecois sur des Matériaux de Pointe (RQMP)

Table S1. The isokinetic prefactor σ_{00}, isokinetic temperature T_{iso}, and the range of measurement temperatures $T\ vs. T_{iso}$ for some lithium-ion conductors. $T_{iso} \in T$ means the isokinetic temperature is in the range of measurement temperature. The three temperature ranges are indicated by their colors, corresponding to Figure 2 of the article.

Material composition	σ_{00} (S·cm^{-1}·K)	$T_{iso}(K)$	$T\ vs. T_{iso}$	Ref.
$Li_6ALa_2Nb_2O_{12}$ (A = Mg, Ca, Sr)	10.01	3436	$T < T_{iso}$	[1]
$Li_3X_3Te_2O_{12}$ (X = Gd, Tb, Er, Lu, Nd)	2.29	2085	$T < T_{iso}$	[2]
$Li_6ALa_2Ta_2O_{12}$ (A = Mg, Ca, Sr, $Sr_{0.5}Ba_{0.5}$, Ba)	-1.25	771	$T < T_{iso}$	[3]
$Li_{10}Ge_{1-x}Sn_xP_2S_{12}$ (x = 0, 0.33, 0.67, 1)	6.91	707	$T < T_{iso}$	[4]
$Li_{10-x}SnP_2S_{12-x}F_x$ (x = 0, 0.05, 0.1, 0.3, 0.6)	5.81	706	$T < T_{iso}$	[5]
$Li_7Sb_xP_{3-x}S_{11-y}I_y$ (y = 0, x = 0; y = 0.5, x = 0.05, 0.1; y = 0.7, x = 0.05)	4.32	580	$T < T_{iso}$	[6]
$Li_{10}Ge_{1-x}Si_xP_2S_{12}$ (x = 0, 0.05, 0.3, 0.7)	496.93	529	$T < T_{iso}$	[7]
$5Li_2S·P_2S_5·(1\text{-}z)GeS_2·zSnSc_2$ (z = 0.2, 0.4, 0.5, 0.75, 1)	2.77	492	$T < T_{iso}$	[8]
$Li_{3+x}V_{1-x}Ge_xO_4$ (x = 0.2, 0.4, 0.55, 0.75, 0.85)	1.93	443	$T < T_{iso}$	[9]
Al-$Li_{7-x}La_3Zr_{2-x}Ta_xO_{12}$ (x = 0.4, 0.5, 0.6, 0.7, 0.8)	2.91	435	$T < T_{iso}$	[10]

$Li_{10}SnP_{2-x}Sb_xS_{12-2.5x}O_{2.5x}$ (x = 0, 0.04, 0.08, 0.12, 0.16, 0.2)	1.81	411	$T < T_{iso}$	[11]
$Li_{4-x}Ge_{1-x}P_xS_4$ (x = 0.2, 0.4, 0.6, 0.7, 0.75)	6.59	1004	$T < T_{iso}$	[12]
$Li_{4-x}Sn_{1-x}As_xS_4$ (x = 0.125, 0.142, 0.166, 0.2, 0.25)	6.04	887	$T < T_{iso}$	[13]
$Li_{10}Ge_{1-x}Sn_xP_2S_{12}$ (x = 0, 0.05, 0.3, 0.7)	4.66	497	$T < T_{iso}$	[7]
$Li_{3+x}Ge_xAs_{1-x}S_4$ (x = 0.286, 0.334, 0.358, 0.4, 0.5)	1.70	457	$T < T_{iso}$	[14]
$Li_5La_3Nb_{2-x}Y_xO_{12}$ (x = 0, 0.05, 0.1, 0.15, 0.2, 0.25)	-3.07	342	$T_{iso} \in T$	[15]
$Li_6BaLa_2Nb_xTa_{2-x}O_{12}$ (x = 0, 0.5, 1, 1.5, 2)	-5.18	330	$T_{iso} \in T$	[16]
$Li_{10}GeP_2S_{12-x}O_x$ (x = 0, 0.3, 0.6)	0.96	294	$T_{iso} \in T$	[17]
$Li_{6.3+x}La_{3-x}Sr_xZr_{1.65}Te_{0.35}O_{12}$ (x = 0, 0.125, 0.15, 0.20, 0.25)	-2.91	285	$T_{iso} \in T$	[18]
$Li_{4-2x}Zn_xGeO_4$ (x = 0.3, 0.4, 0.55, 0.75, 0.85)	-8.35	327	$T_{iso} \in T$	[19]
$Li_6PS_5Cl_{1-x}Br_x$ (x = 0, 0.25, 0.5, 0.75, 1)	-1.80	275	$T_{iso} \in T$	[20]
$Li_{3+x}As_{1-x}Ti_xO_4$ (x = 0.25, 0.4, 0.5, 0.6, 0.75)	-19.75	160	$T > T_{iso}$	[9]

Table S2. The isokinetic prefactor σ_{00}, isokinetic temperature T_{iso}, and the range of measurement temperatures $T\ vs. T_{iso}$ for some proton conductors. $T_{iso} \in T$ means the isokinetic temperature is in the range of measurement temperature. The three temperature ranges are indicated by their colors, corresponding to Figure 2 of the article.

Material composition	Conductivity type	σ_{00} (S·cm^{-1}·K)	$T_{iso}(K)$	$T\ vs. T_{iso}$	Ref
$BaCe_{0.85-x}Zr_xSm_{0.15}O_{3-\delta}$ (0.01 < x < 0.3)	Total	1.50	2121	$T < T_{iso}$	[21]
$BaCe_{0.5}Zr_{0.3}Ln_{0.2}O_{3-d}$ (Ln = Yb, Gd, Sm, Nd, La or Y)	Total	3.21	2404	$T < T_{iso}$	[22]
$BaCe_{0.5}Zr_{0.3}Ln_{0.2}O_{3-d}$ (ln = Y, Dy, Sm, Nd)	Bulk	5.15	2692	$T < T_{iso}$	[23]
$BaCe_{0.8-x}Zr_xY_{0.2}O_3$ (x = 0.2, 0.4, 0.6, 0.8)	Total	0.38	984	$T < T_{iso}$	[24]
$BaZr_{0.9}M_{0.1}O$ (M = Ho, Er, Tm, Yb)	Bulk	0.94	1269	$T < T_{iso}$	[25]
$BaZr_{0.8}M_{0.2}O_3$ (M = Ho, Er, Tm, Yb)	Bulk	0.85	1306	$T < T_{iso}$	[25]
$BaCe_{0.45}Zr_{0.45}MO_3$ (M = In, Sm, Gd, Y)	Total	0.33	1556	$T < T_{iso}$	[26]
$BaCe_{0.8-x}Zr_xY_{0.2}O_3$ (x = 0-0.8)	Bulk	5.69	2062	$T < T_{iso}$	[27]
$BaCe_{0.77-x}Zr_xGd_{0.2}Cu_{0.03}O_{3-\delta}$	Total	10.24	3340	$T < T_{iso}$	[28]
$Ba_{1-x}Sr_xCe_{0.8}Y_{0.2}O_{3-\delta}$	Total	111.28	3900	$T < T_{iso}$	[29]
$BaZr_{0.8-x}Ce_xY_{0.2}O_3$ (x=0,0.2,0.4)	Bulk	0.05	498	$T_{iso} \in T$	[30]
$BaZr_{0.9}Y_{0.1}O_3$	Bulk	1.10	596	$T_{iso} \in T$	[31]

$BaZr_{0.98}M_{0.02}O_3$ (M = Ho, Er, Tm, Yb)	Bulk	4.90E-03	596	$T_{iso} \in T$	[25]
$BaZr_{0.9}Y_{0.1}O_3$	Grain boundary	106.20	643	$T_{iso} \in T$	[31]
$BaZr_{0.85}M_{0.15}O_3$ (M = Ho, Er, Tm, Yb)	Bulk	0.14	823	$T_{iso} \in T$	[25]
$BaCe_{0.8}Y_{0.2}O_3$	Grain boundary	3.44E-03	439	$T_{iso} \in T$	[32]
$BaCe_{0.8}Y_{0.2}O_3$	Bulk	2.00E-05	297	$T > T_{iso}$	[32]

References

1. V. Thangadurai and W. Weppner, J. Am. Ceram. Soc., 2005, 88, 411.
2. E. J. Cussen, T. W. Yip, G. O'Neill and M. P. O'Callaghan, J. Solid State Chem., 2011, 184, 470.
3. R. Murugan, V. Thangadurai and W. Weppner, J. Electrochem. Soc., 2007, 155, A90.
4. T. Krauskopf, S. P. Culver and W. G. Zeier, Chem. Mater., 2018, 30, 1791.
5. Z. Jiang, Z. Li, X. Wang, C. Gu, X. Xia and J. Tu, ACS Appl. Mater. Interfaces, 2021, 13, 30739.
6. M. K. Tufail, L. Zhou, N. Ahmad, R. Chen, M. Faheem, L. Yang and W. Yang, Chem. Eng. J., 2021, 407, 127149.
7. Y. Kato, R. Saito, M. Sakano, A. Mitsui, M. Hirayama and R. Kanno, J. Power Sources, 2014, 271, 60.
8. K. Yang, J. Dong, L. Zhang, Y. Li, L. Wang and J. Stevenson, J. Am. Ceram. Soc., 2015, 98, 3831.
9. A. Rodger, J. Kuwano and A. West, Solid State Ionics, 1985, 15, 185.
10. D. Ma, Material Sciences, 2017, 07, 243.
11. J. Gao, X. Sun, C. Wang, Y. Zhang, L. Yang, D. Song, Y. Wu, Z. Yang, T. Ohsaka and F. Matsumotoc, ChemElectroChem, 2022, 9,

12. R. Kanno and M. Murayama, J. Electrochem. Soc., 2001, 148, A742.
13. G. Sahu, Z. Lin, J. Li, Z. Liu, N. Dudney and C. Liang, Energy & Environmental Science, 2014, 7, 1053.
14. G. Sahu, E. Rangasamy, J. Li, Y. Chen, K. An, N. Dudney and C. Liang, J. Mater. Chem. A, 2014, 2, 10396.
15. S. Narayanan and V. Thangadurai, J. Power Sources, 2011, 196, 8085.
16. Y. Zhong, Q. Zhou, Y. Guo, Z. Li and Y. Qiang, Ionics, 2013, 19, 697.
17. Y. Sun, K. Suzuki, K. Hara, S. Hori, T. A. Yano, M. Hara, M. Hirayama and R. Kanno, J. Power Sources, 2016, 324, 798.
18. X. Shen, Q. Zhang, T. Ning, J. Liu, T. Liu, Z. Luo, Y. He, C. Qin and A. Lu, Solid State Ionics, 2020, 356, 115427.
19. P. Bruce and A. West, J. Solid State Chem., 1982, 44, 354.
20. M. A. Kraft, S. P. Culver, M. Calderon, F. Bocher, T. Krauskopf, A. Senyshyn, C. Dietrich, A. Zevalkink, J. Janek and W. G. Zeier, Journal of the American Chemical Society, 2017, 139,
21. D. Han, N. Hatada and T. Uda, J. Electrochem. Soc., 2016, 163, F470.
22. P. Sawant, S. Varma, B. N. Wani and S. R. Bharadwaj, Int. J. Hydrogen Energy, 2012, 37, 3848.
23. J. Lv, L. Wang, D. Lei, H. Guo and R. V. Kumar, J. Alloys Compd., 2009, 467, 376.
24. D. Han, X. Liu, T. S. Bjørheim and T. Uda, Adv. Energy Mater., 2021, 11, 2003149.
25. R. Kannan, S. Gill, N. Maffei and V. Thangadurai, J. Electrochem. Soc., 2012, 160, F18.
26. J. Lyagaeva, D. Medvedev, E. Filonova, A. Demin and P. Tsiakaras, Scripta Mater., 2015, 109, 34.
27. N. Danilov, E. Pikalova, J. Lyagaeva, B. Antonov, D. Medvedev, A. Demin and P. Tsiakaras, J. Power Sources, 2017, 366, 161.
28. D. A. Medvedev, E. V. Gorbova, A. K. Demin and B. D. Antonov, Russ. J.

Electrochem., 2011, 47, 1404.

29. S. Wang, F. Zhao, L. Zhang, K. Brinkman and F. Chen, J. Alloys Compd., 2010, 506, 263.

30. D. Han, K. Goto, M. Majima and T. Uda, ChemSusChem, 2021, 14, 614.

31. Q. Chen, A. Braun, A. Ovalle, C.-D. Savaniu, T. Graule and N. Bagdassarov, Appl. Phys. Lett., 2010, 97, 041902.

32. Q. Chen, A. Braun, S. Yoon, N. Bagdassarov and T. Graule, J. Eur. Ceram. Soc., 2011, 31, 2657.
